# Supplementary material for: Metabolomic, lipidomic, and N-glycomic analyses of a human cell model of Krabbe disease reveal treatable deficits in glycosylation and serine-ceramide metabolism
Source: bioRxiv. 2026 Aug 13:2026.08.12.744295. Preprint. [Version 1] doi: 10.64898/2026.08.12.744295 (PMC13640690; doi:10.64898/2026.08.12.744295)

bioRxiv preprint doi: https://doi.org/10.64898/2026.08.12.744295; this version posted August 13, 2026. The copyright holder for this preprint (which was not certified by peer review) is the author/funder, who has granted bioRxiv a license to display the preprint in perpetuity. It is made available under a CC-BY 4.0 International license.

Supplementary Figure 1. Glycolysis, glycosylation, and energy balance are perturbed in *GALC* KO cells. Glycolytic metabolite pools' (A) Relative amounts, normalized against the average level of WT cells treated with high glucose, and (B) ^{13}C-glucose-derived labeling. These are the same metabolites, in order, presented in Figure 2A: Glucose-6-phosphate or Fructose-6-phosphate (G6P-F6P), Fructose1,6-bisphosphate (F16BP), 3-phosphoglycerate (3PG), Phosphoenolpyruvate (PEP), and Lactate (Lac). ****$p<0.0001$, **$p<0.01$, *$p<0.05$ by 2-way ANOVA with Tukey's correction for multiple comparisons. Data compiled from n=4 replicates. C) Ratios of the pool sizes of energy-carrying species. The ratio of ATP to ADP and ATP to AMP was significantly lower in *GALC* KO cells compared to WT cells, but only at high glucose levels. **$p<0.01$, *$p<0.05$ by one-way ANOVA with Šidák's correction for multiple comparisons. Data compiled from n=4 replicates. D) Glycosylation metabolite pools' relative amounts, normalized against the average level of WT cells treated with low glucose. Gal1P levels were significantly reduced in *GALC* KO cells. **$p<0.01$ by 2-way ANOVA with Tukey's correction for multiple comparisons. Data compiled from n=4 replicates. E) Psychosine levels in WT and KO cells do not increase with 1mM nor 5mM galactose supplementation. n.s. not significant by 2-way ANOVA with Tukey's correction for multiple comparisons. Data compiled from n=6 replicates.

Supplementary Figure 1

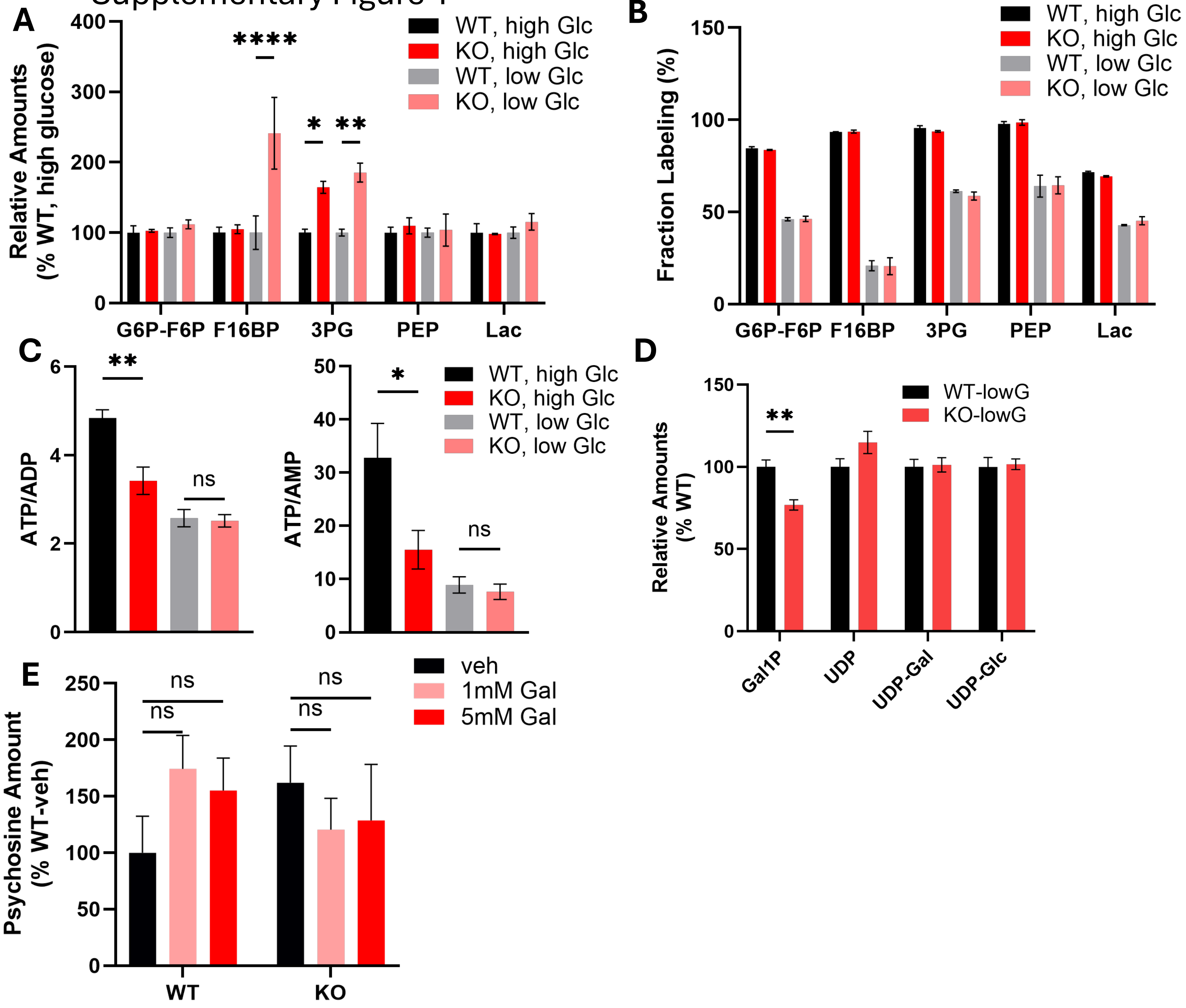

Supplement: Supplementary file 1 [file NIHPP2026.08.12.744295v1-supplement-1.pdf]
